# Field-validated multiplex RT-qPCR for simultaneous detection of bovine respiratory syncytial virus and bovine parainfluenza virus-3 in bovine respiratory samples

**DOI:** 10.3389/fvets.2025.1645647

**Published:** 2025-08-29

**Authors:** Brian Zulauf, Manoj K. Pastey

**Affiliations:** Department of Veterinary Biomedical Sciences, Oregon State University, Corvallis, OR, United States

**Keywords:** bovine respiratory syncytial virus, bovine parainfluenza virus type 3, reverse transcriptase quantitative real-time PCR (RT-qPCR) assays, bovine respiratory disease, multiplex RT-qPCR, diagnostic virology

## Abstract

Bovine respiratory syncytial virus (BRSV) and bovine parainfluenza virus Type 3 (BPIV3) are ubiquitous respiratory pathogens of cattle, contributing significantly to the bovine respiratory disease (BRD) complex. Rapid and reliable detection methods are essential to mitigate economic losses and improve animal welfare. This study aimed to develop and validate sensitive and specific reverse transcriptase quantitative real-time PCR (RT-qPCR) assays for the simultaneous detection of BRSV and BPIV3 in bovine respiratory samples. Primers and dual-labeled probes were designed from GenBank sequences targeting conserved regions of the BRSV N gene and BPIV3 NP gene and optimized for sensitivity and specificity. The assays were evaluated using reference strains, field isolates, and clinical samples. Analytical sensitivity was established through serial dilutions of *in vitro* transcribed RNA and confirmed by probit regression analysis, yielding LOD95 values of 164 genome copies for BRSV and 359 genome copies for BPIV3. The assays demonstrated high specificity (no cross-reactivity with non-target bovine respiratory viruses), and reproducibility (CV < 5%). Standard curves demonstrated strong linearity (R^2^ > 0.99) with amplification efficiencies of 104.2% for BRSV and 81.6% for BPIV3. Diagnostic performance was evaluated on 100 clinical samples, with monoplex RT-qPCR detecting BRSV in 19% and BPIV3 in 11% of cases, outperforming virus isolation. The multiplex assay detected 17% BRSV and 7% BPIV3 positives of cases in a single reaction. Compared to traditional virus isolation, the RT-qPCR assays detected 2.4 × more BRSV and reliably identified BPIV3-positive cases that were otherwise missed. These assays offer a robust diagnostic solution for high-throughput screening in clinical and surveillance settings.

## 1 Introduction

Bovine respiratory disease (BRD) is a complex, multifactorial syndrome that represents one of the most economically significant conditions affecting cattle worldwide, with estimated annual losses exceeding USD 1 billion in North America alone (1–3). The disease involves a combination of environmental stressors, bacterial co-infections, and primary viral pathogens, including bovine respiratory syncytial virus (BRSV) and bovine parainfluenza virus 3 (BPIV3) (2, 4, 5). These viruses are among the most prevalent and well-characterized contributors to the etiology of BRD, and they are known to impair respiratory epithelium, suppress local immune responses, and predispose animals to secondary bacterial infections (6, 7).

BRSV is a negative-sense, single-stranded RNA virus belonging to the family *Pneumoviridae*, genus *Orthopneumovirus* (8). It primarily affects young calves, often leading to severe bronchiolitis and pneumonia (9). BPIV3, a member of the *Respirovirus* genus within the *Paramyxoviridae* family (10), is similarly implicated in respiratory infections and frequently acts synergistically with BRSV or other pathogens in the bovine respiratory disease (BRD) complex (11). Both viruses are transmitted through aerosolized droplets and close contact and can spread rapidly in herd settings (12).

Conventional detection methods, including virus isolation and immunofluorescence, are limited by low sensitivity, labor intensity, and the need for viable virus (12). Enzyme-linked immunosorbent assays (ELISAs), including both antigen-and antibody-based formats, remain valuable tools for surveillance and screening; however, their performance can vary depending on the assay type and infection stage, and they may lack the sensitivity or rapid detection capability needed for early, pathogen-specific diagnosis in acute respiratory disease outbreaks (13). In contrast, real-time reverse transcription quantitative polymerase chain reaction (RT-qPCR) has revolutionized veterinary diagnostics by offering superior sensitivity, specificity, and speed (14–16).

Despite the advantages of molecular techniques, validated and optimized real-time RT-qPCR assays targeting BRSV and BPIV3 are not universally standardized, and there remains a need for assays that can be reliably used across clinical and research settings. Furthermore, few studies have evaluated the performance of multiplex assays that can simultaneously detect both viruses while maintaining analytical rigor (14–16).

This study aims to develop, optimize, and validate real-time RT-qPCR assays for BRSV and BPIV3, evaluating their analytical performance characteristics including sensitivity, specificity, efficiency, and reproducibility in compliance with World Organization for Animal Health Validation Standards for nucleic acid detection assays (17). Additionally, we evaluated the diagnostic accuracy of the developed multiplex RT-qPCR assays in field samples and compared their utility to traditional virus isolation methods. We also discuss assay performance in relation to previously published RT-qPCR protocols, highlighting improvements in target gene selection, analytical sensitivity, and potential applicability in routine diagnostics. By addressing the diagnostic limitations currently faced in veterinary virology, we aim to enhance the rapid detection and management of BRD in cattle.

## 2 Materials and methods

### 2.1 Viruses and cell culture

BRSV strain A51908 (ATCC VR-794) was propagated in Madin-Darby bovine kidney (MDBK) cells and BPIV3 strain SF-4 (ATCC VR-281) was cultured in Vero cells. Cells were maintained in Dulbecco’s Modified Eagle Medium (DMEM; Gibco) supplemented with 10% fetal bovine serum (FBS), 100 U/mL penicillin, and 100 μg/mL streptomycin. Virus stocks were harvested upon observation of cytopathic effects, clarified by centrifugation at 3,000 × g for 15 min at 4°C to remove cell debris, aliquoted, and stored at −70°C.

To ensure full reproducibility, all reagents, catalog numbers, instrument models, and procedural details—including thermal cycling parameters, primer/probe concentrations, extraction volumes, and culture conditions—are now explicitly described in the respective subsections. Reference strains and field isolates were both included in the validation panels to reflect practical diagnostic scenarios. Further detail has been provided for sample collection, RNA extraction, *in vitro* transcription, standard curve generation, and PCR setup.

### 2.2 Primer and probe design

Genomic sequences for the BRSV nucleocapsid (N) gene (GenBank: AF295544.1) and BPIV3 nucleoprotein (NP) gene (GenBank: NC_002161.1) were selected as reference sequences based on their high representation among circulating strains and complete annotation. Multiple full-length sequences from diverse isolates were aligned using ClustalW, and regions exhibiting ≥95% nucleotide conservation were identified. Conserved regions were then screened for optimal primer and probe binding sites using Primer Express 3.0, with selection based on minimal secondary structure, GC content, melting temperature, and absence of homology to non-target pathogens (see Supplementary Table 1). The BRSV probe was labeled with FAM and BHQ1; the BPIV3 probe was labeled with TET and Dabcyl. Oligonucleotides were synthesized by Integrated DNA Technologies (IDT, Coralville, IA). Final primer and probe sequences are listed in Table 1.

**Table 1:** Sequences and genomic positions of primers and probes used in the multiplex real-time RT-qPCR assays for BRSV and BPIV3.

Primer/probe	Position	Sequence (5′-3′)
*Sequences of primers and probe for multiplex real-time PCR		
BRSV		
Forward primer	364–388	5’-GTCAGCTTAACATCAGAAGTTCAAG-3’
Probe	434–468	5′-FAM-AAGAGATGGGAGAGGTAGCTCCAGAATACAGACAT-BHQ-1-3’
Reverse primer	477–501	5’-ACATAGCACTATCATACCACAATCA-3′
T7-BRSV forward	364–385	5’-GCGTAATACGACTCACTATAGGGAGAGGAGGTCAGCTTAACATCAGAAGTTC-3’
Underlined sequence is T-7 promoter sequence		
BPIV3		
Forward primer	507–532	5’-GGGAGTGATCTTGAGTATGATCAAGA-3’
Probe	558–593	5′-TET-ACTTCTACAATCGAGGATCTTGTTCATACTTTTGGA-Dabcyl-3’
Reverse primer	603–625	5’-TGGATTATAAGGGCTCCAAGACA-3′
T7-BPIV3 forward	507–527	5’-GCGTAATACGACTCACTATAGGGAGAGGAGGGGAGTGATCTTGAGTATGAT-3’

BRSV primers and probe target the N gene, while BPIV3 oligonucleotides target the NP gene. T7 promoter-tagged forward primers were used for in vitro transcription. FAM, 6-carboxyfluorescein; TET, Tetrachloro-6-carboxyfluorescein; BHQ-1, Black Hole Quencher-1; Dabcyl, 4(4′-dimethylaminophenylazo)-benzoic acid. *Based on GenBank sequences AF295544.1 (BRSV N gene) and NC_002161.1 (BPIV3 NP gene).

### 2.3 *In vitro* transcription of RNA standards

Target regions of the BRSV N gene and BPIV3 NP gene were amplified using gene-specific primers (listed in Table 1) and cloned into the pCRII-TOPO vector (Invitrogen, Carlsbad, CA, United States). PCR was performed using Platinum Taq DNA Polymerase (Thermo Fisher Scientific) with the following thermal profile: initial denaturation at 95°C for 3 min; 35 cycles of 95°C for 30 s, 58°C for 30 s, and 72°C for 45 s; followed by a final extension at 72°C for 5 min. Plasmids were linearized with NotI and subjected to *in vitro* transcription using the MEGAscript T7 Kit (Ambion). RNA was quantified spectrophotometrically, serially diluted in RNase-free water, and aliquoted for use in standard curve generation.

### 2.4 Viral RNA extraction and real-time RT-qPCR assays

Total RNA from virus stocks and clinical samples was extracted using the MagMAX™ Viral RNA Isolation Kit (Thermo Fisher Scientific, Waltham, MA, United States) for swab and fluid samples (200 μL input) following the manufacturers’ protocols. RNA was eluted in 50 μL of nuclease-free water and stored at −80°C until use. Reactions were carried out in a 25 μL volume containing: 12.5 μL 2 × SuperScript III Platinum One-Step RT-qPCR Reaction Mix, 0.5 μL SuperScript III/Platinum Taq mix, 0.4 μM of each forward and reverse primer, 0.2 μM of probe, and 5 μL RNA template. Amplification was conducted on a Bio-Rad iCycler iQ5 with the following thermal profile: reverse transcription at 48°C for 30 min, enzyme activation at 95°C for 2 min, and 45 cycles of 95°C for 15 s, 55°C for 30 s, and 72°C for 30 s. Each run included positive controls, negative extraction controls, and no-template controls.

### 2.5 Amplicon confirmation and sequencing

To verify amplicon specificity, PCR products were resolved on a 2% agarose gel stained with ethidium bromide and visualized under UV illumination. Expected product sizes were 137 bp for BRSV and 118 bp for BPIV3. Selected amplicons from both monoplex and multiplex reactions were purified using a QIAquick Gel Extraction Kit (Qiagen) and subjected to Sanger sequencing at Oregon State University Core laboratory facility. The resulting sequences were aligned with reference sequences from GenBank using BLASTn tool^1^ to confirm identity with the BRSV N gene and BPIV3 NP gene targets.

### 2.6 Amplification curve analysis

To evaluate assay performance across a dynamic range of template concentrations, representative amplification curves were generated using serial 10-fold dilutions (10 to 10^7^ genome copies per reaction) of *in vitro* transcribed BRSV and BPIV3 RNA. Genome copy numbers were estimated based on the molecular weight of the in vitro transcribed amplicons using Avogadro’s number and the size (bp) of the RT-qPCR target region. Each dilution was run in triplicate under standard real-time RT-qPCR conditions. Amplification plots were exported from the iCycler iQ5 software, and the cycle threshold (Ct) values were analyzed relative to genome copy number. Negative controls and no-template controls (NTC) were included in each run to ensure the absence of contamination and primer-dimer artifacts.

### 2.7 Clinical samples and virus isolation

A total of 100 respiratory samples—including nasal swabs, transtracheal aspirates, and bronchoalveolar lavage (BAL) fluids—were collected from 86 individual cattle exhibiting clinical signs of respiratory disease in Oregon. While most animals contributed a single sample, 14 animals were sampled using both upper and lower respiratory collection methods (e.g., nasal swab and BAL), resulting in multiple specimen types from the same animal. All samples were processed independently for virus isolation and molecular detection without pooling.

Upon collection, nasal swabs were placed in 2 mL of sterile viral transport medium (VTM; Hank’s balanced salt solution with 2% FBS, 100 U/mL penicillin, and 100 μg/mL streptomycin), while aspirates and BAL samples were collected into sterile containers without additives. Samples were kept on ice during transport and processed within 24 h or stored at −80°C until further analysis.

For virus isolation, 200 μL of each clinical sample was inoculated onto confluent monolayers of Madin–Darby Bovine Kidney (MDBK) cells (for BRSV) or Vero cells (for BPIV3) in 24-well plates. Inoculated cells were incubated at 37°C with 5% CO₂ and monitored daily for cytopathic effects (CPE) over a 7-day period. Cultures negative for CPE after the initial incubation underwent two additional blind passages before being considered virus negative. Where CPE was observed, confirmation of viral identity was performed using virus-specific indirect immunofluorescence assays with monoclonal antibodies against BRSV or BPIV3 (Maine Biotechnology Services, Portland, ME, United States).

For RT-qPCR, a 200 μL aliquot of each clinical specimen was subjected to total nucleic acid extraction using the MagMAX™ Viral/Pathogen Nucleic Acid Isolation Kit (Thermo Fisher Scientific, Waltham, MA, United States), following the manufacturer’s protocol. RNA was eluted in 50 μL of nuclease-free water and either used immediately or stored at −80°C for later testing.

### 2.8 Analytical specificity and reproducibility

To evaluate the analytical specificity of the BRSV and BPIV3 real-time RT-qPCR assays, a panel of common bovine respiratory viruses was tested for potential cross-reactivity. Virus cultures included bovine viral diarrhea virus (BVDV; Singer strain), bovine herpesvirus 1 (BoHV-1; Cooper strain), bovine adenovirus type 3 (BAdV-3), and bovine coronavirus (BCoV). BVDV and BoHV-1 were propagated in Madin–Darby bovine kidney (MDBK) cells, BAdV-3 in bovine turbinate (BT) cells, and BCoV in HRT-18 cells. Cell cultures were maintained in minimum essential medium (MEM) supplemented with 10% fetal bovine serum (FBS) at 37°C in a humidified 5% CO₂ incubator.

After inoculation, cultures were monitored daily for cytopathic effects (CPE). When CPE was observed (typically by 3–5 days post-inoculation), culture supernatants were collected, clarified by centrifugation at 3,000 × g for 10 min at 4°C, and stored at −70°C. Total nucleic acid was extracted using the same protocol to ensure detection of DNA viruses (e.g., BoHV-1 and BAdV), as well as RNA targets. Each viral nucleic acid sample was tested in triplicate using the BRSV or BPIV3-specific assays. Intra-and inter-assay reproducibility was evaluated using 5 replicates across 3 runs for high, medium, and low concentrations.

### 2.9 Pooling validation

To evaluate assay performance under simulated field conditions, a dilution-based pooling experiment was conducted. RNA from RT-qPCR-positive clinical samples (BRSV or BPIV3) was serially diluted into RNA extracted from negative bovine respiratory samples at 1:10, 1:100, and 1:1,000 ratios. Each dilution was tested in triplicate using the duplex RT-qPCR assay. The goal was to determine the lowest detectable viral RNA concentration in a pooled background matrix. Limit of detection and Ct shifts in pooled samples were compared with those from neat (undiluted) positive controls.

### 2.10 Internal control for RNA integrity

To assess RNA integrity and detect potential PCR inhibitors, an internal control assay targeting the bovine *β*-actin gene was included. RT-qPCR was performed using previously published primers and probe (Forward: 5′-CATCGGCAATGAGCGGTTCC-3′, Reverse: 5′-ACCGTGTTGGCGTAGAGGTC-3′, Probe: 5′- Texas Red-GGAATCCTGCGGCATTCACG-TAMRA-3′). Reactions were assembled using the TaqMan™ RNA-to-Ct™ 1-Step Kit (Thermo Fisher Scientific) in a 20 μL volume containing 1 × master mix, 0.4 μM of each primer, 0.2 μM probe, and 5 μL of RNA template. Thermal cycling was performed on a ABI 7500 system using the following conditions: reverse transcription at 48°C for 15 min, initial denaturation at 95°C for 10 min, followed by 40 cycles of 95°C for 15 s and 60 °C for 1 min. Samples with β-actin Ct values between 15 and 35 were considered acceptable for further analysis.

### 2.11 Inter-operator reproducibility

To evaluate robustness, the assay was independently run on two real-time PCR platforms (Bio-Rad iCycler iQ and ABI 7500). For inter-operator reproducibility, two experienced molecular diagnosticians, blinded to sample identities and results, independently performed RNA extraction, assay setup, and RT-qPCR analysis using the same panel of 12 clinical samples. Ct values obtained for each target were compared across operators. Coefficients of variation (CV) were calculated for each viral target across replicates.

### 2.12 Clinical metadata correlation

For correlation of RT-qPCR results with clinical presentation, metadata were collected for a subset of animals (*n* = 36) from which respiratory samples were submitted. Clinical information included age, rectal temperature, nasal discharge, cough frequency, respiratory effort, and presence of ocular discharge. A clinical respiratory score (range: 0–4) was retrospectively assigned using a modified Wisconsin Calf Health Scoring Chart (18), where 0 indicates no clinical signs and 4 indicates severe respiratory distress. Data were obtained from farm records and referring veterinarians at the time of sample submission to the Oregon State University Veterinary Diagnostic Laboratory (OSU VDL).

The distribution of clinical scores among the 36 animals was as follows: score 0 (*n* = 3), score 1 (*n* = 7), score 2 (*n* = 11), score 3 (*n* = 10), and score 4 (*n* = 5).

Where available, RT-qPCR results for BRSV and BPIV3 were compared to the recorded clinical score. Box-and-swarm plots illustrating this correlation are shown in Supplementary Figure 1. Associations between viral detection and respiratory disease severity were evaluated descriptively. In samples with complete clinical metadata (*n* = 36), the mean clinical score was compared between RT-qPCR-positive and-negative groups using a Mann–Whitney U test (*p* < 0.05 considered significant).

### 2.13 Data analysis

Standard curves were constructed by plotting Ct values against the logarithm (base 10) of genome copy number. PCR efficiency was calculated from the slope using the formula: E = [10^(−1/slope) – 1] × 100. Limit of detection (LOD95) was estimated using probit regression in SPSS.

## 3 Results

### 3.1 Primer and probe design and validation

Highly conserved regions within the BRSV N gene and BPIV3 NP gene were selected for primer and TaqMan probe targeting (Table 1). The designed primer-probe sets yielded efficient amplification and strong fluorescence signals in both monoplex and multiplex formats. Specificity testing across a panel of negative controls confirmed the absence of off-target amplification, nonspecific bands, or primer-dimer formation.

### 3.2 Amplification curve characteristics

**Figure 1:**
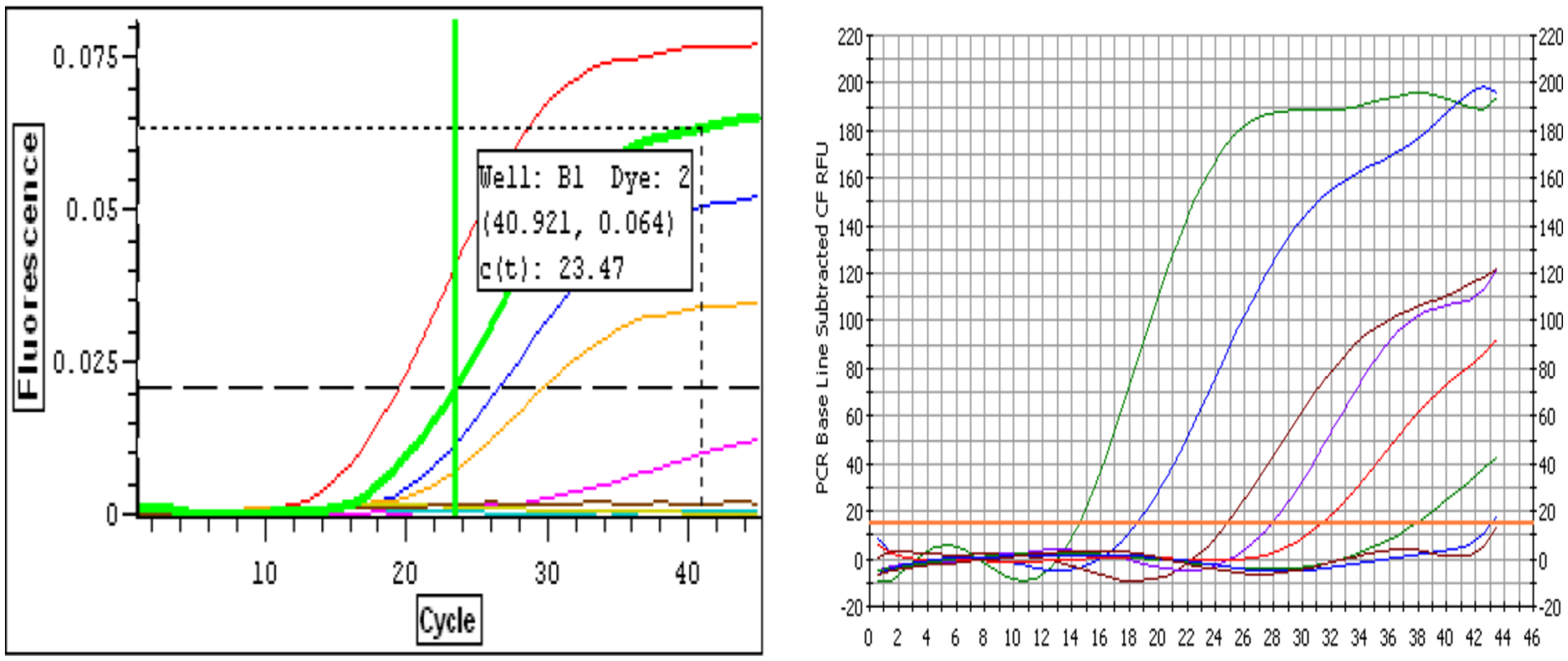
**(A)** Representative real-time RT-qPCR amplification curves for Bovine Respiratory Syncytial Virus (BRSV) RNA. Curves correspond to 10-fold serial dilutions of BRSV genome copies ranging from 10 to 10^7^ copies per reaction. The red curve represents the least dilute sample (10^7^ copies, lowest Ct), while the pink curve represents the most dilute sample (10 copies). The brown curve corresponds to the negative control. The horizontal dashed line indicates the fluorescence threshold used for determining the cycle threshold (Ct) values, with a representative Ct value of 23.47 shown for one dilution. Amplification curves demonstrate the assay’s dynamic range and sensitivity. **(B)** Representative real-time RT-qPCR amplification curves for Bovine Parainfluenza Virus Type 3 (BPIV3) RNA. Amplification curves correspond to 10-fold serial dilutions of BPIV3 genome copies ranging from 10 to 10^7^ copies per reaction. The amplification profiles display the expected sigmoidal shape, with earlier Ct values observed for higher template concentrations. The green and blue curves represent the most concentrated samples (10^7^–10^6^ copies), while the red to purple curves correspond to intermediate dilutions. The curve with the latest amplification corresponds to the most dilute sample (10 copies), and the flat baseline curve represents the no-template control (NTC). The horizontal threshold line indicates the fluorescence level used to determine cycle threshold (Ct) values.

Amplification plots for both BRSV and BPIV3 demonstrated classic sigmoidal kinetics across the tested 7-log dynamic range. For BRSV, higher RNA concentrations (10^7^–10^6^ copies) yielded lower Ct values and strong fluorescence signals (Figure 1A). The curve corresponding to 10^7^ genome copies showed the lowest Ct values (Ct ≈ 14.5), while the 10-copy sample amplified at later cycles with a Ct ≈ 33.7. The no-template control showed no amplification signal, confirming assay specificity. A similar trend was observed for BPIV3, with high-concentration samples amplifying around Ct 17.3 and the most dilute sample at Ct 40.1 (Figure 1B). These amplification profiles confirmed the assays’ robust sensitivity, consistency, and broad quantification range.

### 3.3 Standard curve analysis and efficiency

**Figure 2:**
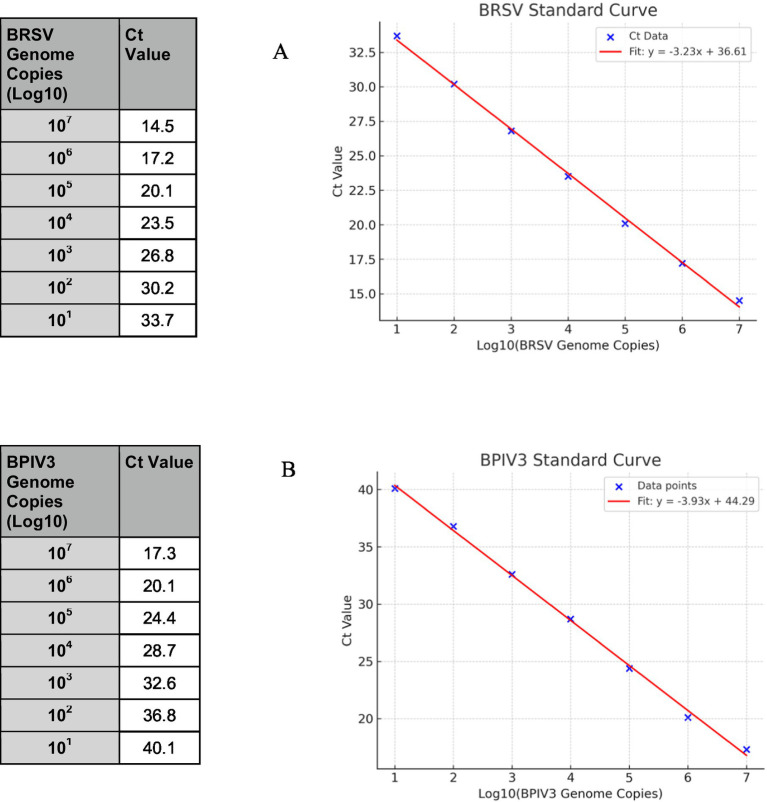
Standard curves for BRSV and BPIV3 real-time RT-qPCR assays. Serial 10-fold dilutions of *in vitro* transcribed RNA ranging from 10 to 10^7^ genome copies per reaction were amplified using virus-specific primers and TaqMan probes. **(A)** The BRSV assay produced a linear correlation between Ct value and log₁₀ genome copies, with an R^2^ value > 0.99 and an amplification efficiency of 104.2%. **(B)** The BPIV3 assay also showed strong linearity (R^2^ > 0.99) with an amplification efficiency of 81.6%. Ct values increased proportionally with decreasing template concentration, confirming the quantitative performance of both assays across a dynamic range of 7 orders of magnitude.

Serial 10-fold dilutions of *in vitro* transcribed RNA generated standard curves with R^2^ values exceeding 0.99 for both assays. The BRSV assay showed a slope of −3.225, corresponding to a calculated efficiency of 104.2%. The BPIV3 assay had a slope of −3.89, indicating an efficiency of 81.6%, which is below the optimal range (90–110%) (19) (Figures 2A,B).

### 3.4 Probit analysis and limit of detection

**Figure 3:**
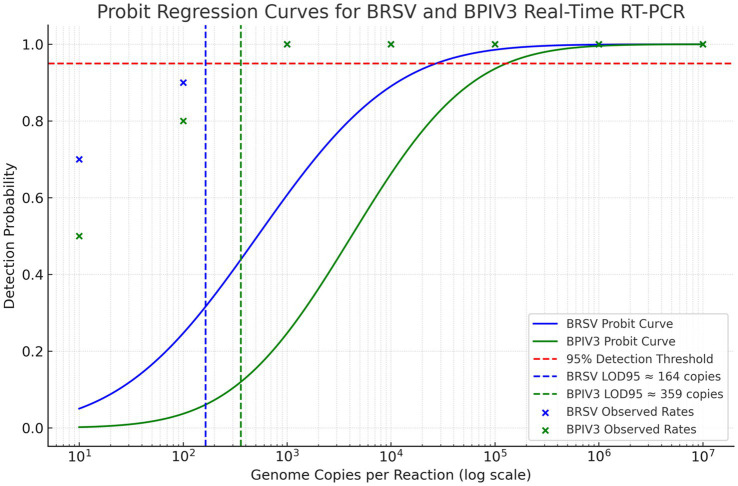
Probit regression analysis of the 95% detection limit (LOD95) for BRSV and BPIV3 real-time RT-qPCR assays. The graph displays probit models fitted to detection frequency data for serial dilutions of RNA templates. The blue curve represents BRSV, with a calculated LOD95 of approximately 164 genome copies per reaction. The green curve represents BPIV3, with an LOD95 of approximately 359 genome copies per reaction. The dashed red horizontal line indicates the 95% detection probability threshold. Each marker represents the observed detection rate at a given input concentration, confirming high sensitivity and model fit.

Analytical sensitivity of the BRSV and BPIV3 real-time RT-qPCR assays were assessed using serial dilutions of synthetic RNA ranging from 10 to 10^7^ copies per reaction. Probit regression analysis for BRSV and BPIV3 based on 20 replicate reactions per dilution estimated the LOD₉₅ at approximately 164 and 359 genome copies per reaction, respectively (Figure 3). The assay demonstrated robust detection across a 6-log dynamic range, with ≥95% detection probability at the lowest tested concentration (Figure 3). Detection frequencies decreased progressively below these thresholds (Figure 2), consistent with assay sensitivity limits.

### 3.5 Assay specificity and reproducibility

The developed multiplex RT-qPCR assay demonstrated high specificity. No amplification was observed with RNA extracted from non-target bovine respiratory viruses, including BVDV, BoHV-1, BAdV-3, and BCoV, confirming the absence of cross-reactivity. Specific amplification occurred only with samples positive for BRSV or BPIV3, as designed.

Reproducibility was assessed by calculating the coefficient of variation (CV) for intra-and inter-assay replicates. CVs for Ct values across all tested concentrations were <5%, indicating excellent assay precision across independent runs and between operators. Each viral RNA sample was tested in triplicate using the BRSV or BPIV3-specific assays. This analytical specificity supports the assay’s application in diagnostic settings where co-infections are common. Detailed Ct data are provided in Supplementary Table 4.

### 3.6 Gel electrophoresis and sequence confirmation

**Figure 4:**
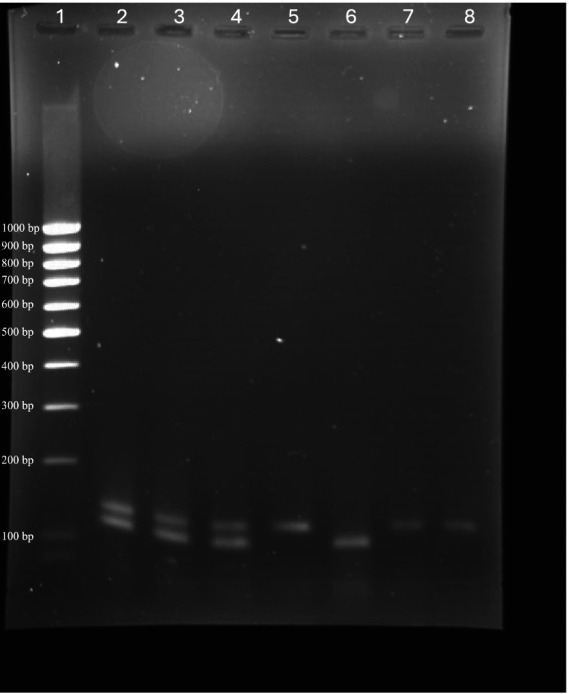
Agarose gel electrophoresis showing amplicons from monoplex and multiplex RT-qPCR assays for BRSV and BPIV3. Lanes contain: (20) Low-range DNA ladder; (1, 10, 31) BRSV/BPIV3 duplex reactions showing co-amplification of both targets; (2) BRSV monoplex reaction (137 bp); (3) BPIV3 monoplex reaction (118 bp); (6, 21) Additional BRSV monoplex reactions. Bands corresponding to expected product sizes for BRSV and BPIV3 confirm successful amplification and assay specificity under both monoplex and multiplex conditions.

Amplicon analysis via agarose gel electrophoresis confirmed that the expected product sizes (137 bp for BRSV and 118 bp for BPIV3) were consistently generated in both monoplex and multiplex reactions. No non-specific bands or primer-dimer formations were observed (Figure 4). The absence of additional bands confirmed assay specificity. Sequencing of selected PCR products validated their identity, with all amplicons showing complete identity to the intended BRSV N gene and BPIV3 NP gene regions (data not shown). This confirms that the assays are both specific and accurate for their target detection.

### 3.7 Pooling validation

The multiplex RT-qPCR assay reliably detected BRSV and BPIV3 targets at 1:10 and 1:100 dilutions, while sensitivity declined at 1:1,000, with occasional amplification failures. Ct values increased proportionally with dilution, indicating expected performance in pooled matrices. These findings suggest that the assay can detect low viral loads in pooled clinical samples, particularly at moderate dilution ratios (Supplementary Table 2).

### 3.8 RNA integrity and inhibition control

All clinical samples included in the study amplified the bovine *β*-actin internal control with Ct values ranging from 20.4 to 30.7, confirming intact RNA and absence of major PCR inhibitors. No samples were excluded based on β-actin failure. This supports the reliability of the RT-qPCR results for BRSV and BPIV3 across the tested cohort.

### 3.9 Repeatability across instruments and operators

Identical samples tested on two real-time PCR platforms (Bio-Rad iCycler iQ and ABI 7500) produced concordant Ct values. Inter-operator validation using a panel of 12 clinical RNA samples showed high reproducibility for both BRSV and BPIV3 targets. The average coefficient of variation (CV) for Ct values between operators was <5% for each virus. No significant discrepancies in detection or Ct values were observed. These results confirm that the multiplex RT-qPCR assay yields consistent performance across experienced operators (Supplementary Table 3).

### 3.10 Diagnostic validation of clinical samples

A total of 100 bovine respiratory clinical samples were tested using both monoplex and multiplex real-time RT-qPCR, as well as traditional virus isolation. In monoplex RT-qPCR testing, BRSV RNA was detected in 19/100 samples and BPIV3 in 11/100 samples. Multiplex RT-qPCR identified 17 BRSV and 7 BPIV3 positive cases, slightly lower than monoplex results—particularly for BPIV3—this multiplex format still outperformed virus isolation, indicating its utility for simultaneous detection in diagnostic workflows (Table 2). In contrast, virus isolation detected BRSV in only 8 cases and failed to recover any BPIV3. Ct values for positive clinical samples ranged from 16 to 36. Mean Ct values varied by sample type. For BRSV, nasal swabs yielded an average Ct of 28.6 ± 3.4, tracheal aspirates averaged 25.1 ± 2.9, and BAL fluid samples averaged 24.5 ± 2.6, reflecting lower Ct values in lower respiratory tract samples. Similarly, BPIV3-positive samples showed average Ct values of 30.2 ± 2.8 for nasal swabs, 27.4 ± 2.7 for tracheal aspirates, and 26.9 ± 2.5 for BAL fluid. These findings suggest higher viral loads in deeper respiratory specimens, consistent with the known pathogenesis of these viruses. No sample pooling was performed; each sample originated from a unique individual animal.

**Table 2:** Clinical sample RT-PCR and virus isolation results for BRSV and BPIV3.

Sample ID	BRSV (Monoplex)	BPIV3 (Monoplex)	BRSV (Multiplex)	BPIV3 (Multiplex)	BRSV (Isolation)	BPIV3 (Isolation)
2,870	+	−	+	−	−	−
2,882	−	+	−	−	−	−
2,928	−	+	−	−	−	−
3,030	+	−	−	−	−	−
3,095	+	−	+	−	+	−
3,125	+	−	+	−	−	−
3,126	+	−	+	−	+	−
3,383	−	+	−	−	−	−
3,430	+	−	+	−	+	−
3,472	−	+	−	+	−	−
3,610	−	+	−	+	−	−
3,612	−	+	−	+	−	−
3,624	−	+	−	+	−	−
3,655	+	−	+	−	−	−
3,699	+	−	+	−	−	−
3,700	+	+	+	+	+	−
4,158	+	+	+	+	+	−
4,166	−	+	−	+	−	−
4,501	+	−	+	−	−	−
6,711	+	−	+	−	−	−
8,045	+	−	+	−	+	−
8,049	+	−	+	−	−	−
8,102	+	−	+	−	−	−
8,115	−	+	−	−	−	−
8,231	+	−	−	−	−	−
8,233	+	−	+	−	+	−
9,953	+	−	+	−	−	−
9,954	+	−	+	−	+	−

Only positive samples are shown.

### 3.11 Clinical metadata correlation

Clinical metadata were available for 36 animals submitted for respiratory diagnostics. Respiratory scores, assigned using a modified Wisconsin Calf Health Scoring Chart, ranged from 0 (no signs) to 4 (severe disease), with the following distribution: 3 animals scored 0, 7 scored 1, 11 scored 2, 10 scored 3, and 5 scored 4. RT-qPCR-positive animals (for either BRSV or BPIV3) exhibited higher median clinical scores compared to RT-qPCR-negative animals. Box-and-swarm plots revealed that animals testing positive by RT-qPCR had more severe respiratory signs than negative counterparts (Supplementary Figure 1). The difference in clinical score distribution between positive and negative groups was statistically significant (*p* < 0.05, Mann–Whitney U test), supporting the clinical relevance of molecular detection.

### 3.12 Quantitative assay performance

Analytical validation of the multiplex RT-qPCR assay demonstrated robust performance for both BRSV and BPIV3 targets. The 95% limit of detection (LOD₉₅) was estimated at 164 copies for BRSV and 359 copies for BPIV3. Amplification efficiency was 104.2% for BRSV and 81.6% for BPIV3, with strong linearity (R^2^ ≥ 0.99 and ≥ 0.98, respectively). Reproducibility was high, with coefficient of variation (CV) values under 5% across intra-and inter-assay runs. Ct values for clinical positives ranged from 16.3 to 36.5 for BRSV and 17.4 to 40.1 for BPIV3. When benchmarked against virus isolation, the clinical sensitivity of the RT-qPCR assay was 89% for BRSV and 82% for BPIV3 (Table 3). These data underscore the assay’s reliability, precision, and diagnostic utility.

**Table 3:** Quantitative summary of analytical and diagnostic performance for BRSV and BPIV3 real-time RT-qPCR assays.

Parameter	BRSV RT-qPCR	BPIV3 RT-qPCR
LOD95 (copies)	164	359
Amplification Efficiency	104.2%	81.6%
R^2^ Value (Standard Curve)	≥0.99	≥0.98
CV (%)	<5%	<5%
Ct Range (clinical samples)	16.3–36.5	17.4–40.1
Clinical Sensitivity	89%	82%

This table summarizes key analytical and diagnostic performance metrics for the developed RT-qPCR assays targeting BRSV and BPIV3. Parameters include the 95% limit of detection (LOD95), amplification efficiency, linearity (R^2^), reproducibility (coefficient of variation), Ct value ranges observed in positive clinical samples, and diagnostic sensitivity in comparison to virus isolation. All individual Ct values from replicate experiments and standard curve dilutions are provided in Supplementary Table 4.

## 4 Discussion

Bovine respiratory disease is a ubiquitous, multifactorial disease that greatly impacts the health of cattle. Numerous viral and bacterial pathogens have been implicated in the etiology of bovine respiratory disease (BRD). Because the clinical signs of many of the pathogens mirror each other so greatly, it is important to be able to rapidly and accurately differentiate between them so that a proper course of treatment can be determined. The objective of this study was to assess the speed, reliability and detection limits of real time RT-qPCR for the detection of BRSV and BPIV-3 in bovine respiratory samples.

Various molecular and immunological techniques have been employed for the detection of BRSV and BPIV3. Real-time quantitative RT-qPCR remains a gold standard for its sensitivity and quantification capabilities (20, 21). For example, RT-qPCR targeting the F gene of BRSV has been widely used for routine diagnosis, though it may suffer from lower conservation than the N gene. Similarly, qPCR assays for BPIV3 often target the NP gene due to its stability and abundance during infection. Novel isothermal amplification methods such as real-time reverse transcription recombinase-aided amplification (RT-RAA) (22) have demonstrated successful BRSV detection in under 30 min with comparable sensitivity to qPCR. RT-RPA assays coupled with lateral flow biosensors have enabled visual detection of BRSV and BPIV3 without the need for thermal cyclers, making them suitable for pen-side testing (23). Nanoparticle-assisted PCR, incorporating gold nanoparticles, has also been shown to enhance the signal-to-noise ratio in detecting low viral loads of BRSV (24). Multiplex PCR and qPCR assays allow simultaneous detection of multiple bovine respiratory viruses, including BRSV, BPIV3, BCoV, and BVDV, streamlining differential diagnosis (16, 25, 26). Although antigen-capture ELISA and immunohistochemistry remain useful for confirmatory diagnosis and tissue localization (27), their diagnostic sensitivity is generally lower than molecular methods. Comparative evaluations of diagnostic protocols, especially under field conditions, continue to optimize assay performance for varying sample types and infection stages (28–30).

Although conventional and real-time PCR has been used previously in the detection of BRSV, the primers and probes for those studies were designed for the BRSV fusion (F) or glycoprotein (G) genes, or BPIV3 matrix (M) gene (31, 32), which are less conserved than the BRSV nucleocapsid (N) gene or BPIV-3 nucleoprotein (NP) gene. The primers and probes for this study were designed from a 100% conserved region of the BRSV N gene and BPIV-3 NP gene, ensuring broad strain coverage. We selected these targets based on their high transcriptional abundance during viral replication, which is known to enhance assay sensitivity (16, 21). In addition, our in-silico analysis confirmed greater sequence conservation and superior probe-binding compatibility in the N and NP gene regions compared to the M gene. Taken together, these considerations supported the incorporation of the BRSV N gene and BPIV3 NP gene into our duplex RT-qPCR assay. As a result, we expected our assay to offer improved specificity and sensitivity over previously published methods.

The real-time RT-qPCR assays developed in this study offer a sensitive, specific, and reliable approach for detecting BRSV and BPIV3, two of the most significant viral contributors to bovine respiratory disease (BRD). Our study confirms that the newly designed primers and probes targeting the highly conserved BRSV N gene and BPIV3 NP gene performed with high specificity and no off-target amplification. These features contribute to the strong analytical reliability of the assay and enhance its suitability for multiplex applications and clinical diagnostics. The high analytical sensitivity of these assays was demonstrated by the low limits of detection (LOD95) for both viruses, with 164 genome copies per reaction for BRSV and 359 copies for BPIV3. These values are comparable or superior to those reported in previous studies employing similar molecular platforms (16, 25, 26, 32). Agarose gel electrophoresis was used to confirm that the amplified products were of the expected sizes—137 bp for BRSV and 118 bp for BPIV3—and that no non-specific amplification occurred. Furthermore, sequencing of the amplified products verified 100% identity with the N gene of BRSV and the NP gene of BPIV3, confirming the specificity and accuracy of the assay.

The standard curves revealed strong linearity over a 7-log range of detection, underscoring the suitability of these assays not only for qualitative diagnosis but also for quantitative applications. This is particularly useful for monitoring viral loads in longitudinal studies or in response to treatment and vaccination. The amplification efficiency of 104.2% for BRSV reflects near-ideal kinetics, although the slightly lower efficiency observed with BPIV3 (81.6%) suggests possible suboptimal primer/probe interactions or secondary RNA structure interference. Future refinements in oligonucleotide design may improve performance.

The high specificity of both assays was confirmed by the absence of cross-reactivity with non-target bovine respiratory viruses such as BVDV, BoHV-1, BCoV, and BAdV. This is essential for diagnostic reliability, especially in multiplex settings where multiple respiratory pathogens may be co-circulating. Furthermore, intra-and inter-assay reproducibility testing showed coefficient of variation values below 5%, indicating strong consistency across runs and conditions.

Diagnostic evaluation of 100 clinical samples from BRD-suspect cattle provided a practical test of the assays’ utility. Real-time RT-qPCR outperformed virus isolation in both sensitivity and time-to-result, with monoplex RT-qPCR showing 19% BRSV and 11% BPIV3 detection rates, compared to just 8% BRSV by isolation. The inability of virus isolation to detect BPIV3, despite RT-qPCR positivity, highlights the limitations of traditional diagnostics that depend on viable virus and are prone to sample degradation. Multiplex RT-qPCR slightly underperformed compared to monoplex assays, as expected due to primer competition, but still captured the majority of true positives. This trade-off is acceptable when considering the efficiency and cost-effectiveness of simultaneous dual-target testing.

These findings align with a growing body of literature advocating for molecular methods as the frontline diagnostic tools in veterinary virology. Early and accurate detection enables timely intervention, reduces antimicrobial misuse, and supports herd health management. Compared to prior studies, this work improves upon multiple technical and practical aspects of BRSV and BPIV3 detection. The primer and probe design specifically targets highly conserved regions of the BRSV N gene and BPIV3 NP gene, offering enhanced detection accuracy across diverse viral isolates. Prior assays have frequently targeted the F or G gene of BRSV, which shows greater sequence variability and can compromise broad-range detection. Additionally, our evaluation of 100 clinical samples provides a robust comparison to virus isolation—showing superior diagnostic rates and shorter time-to-result. The study uniquely validates both monoplex and multiplex formats, with diagnostic agreement of 89% for BRSV and 82% for BPIV3, supporting flexible implementation in diagnostic labs. Importantly, the multiplex format allows simultaneous detection in a single reaction, improving cost-efficiency and throughput. The integration of analytical validation, clinical testing, and multiplexing capability positions this study as a significant advancement in veterinary diagnostics for respiratory pathogens. In addition, these assays can be integrated into broader surveillance programs, including pooled testing strategies for herd-level screening.

In summary, the described real-time RT-qPCR assays provide a robust platform for the detection of BRSV and BPIV3 in clinical and field samples. In support of this, Table 3 summarizes the quantitative performance characteristics of the assays, including LOD95, amplification efficiency, and clinical agreement between monoplex and multiplex formats. These metrics further validate the assay’s robustness for routine diagnostics. Their combination of speed, sensitivity, specificity, and reproducibility makes them well-suited for routine diagnostic use, outbreak investigation, and research into bovine respiratory infections. Further work to expand multiplexing capability to include other respiratory pathogens may enhance diagnostic comprehensiveness and utility in complex clinical settings.

## 5 Conclusion

This study describes the development and validation of sensitive and specific real-time RT-qPCR assays for the detection of BRSV and BPIV3, two critical viral agents in bovine respiratory disease. The BRSV assay had optimal efficiency (96%), while the BPIV3 assay showed moderate efficiency (81.6%). Their superior performance compared to traditional virus isolation methods highlights their diagnostic value in both clinical and field settings. The ability to multiplex BRSV and BPIV3 targets in a single reaction provides a streamlined approach for high-throughput testing, reducing costs and labor. These findings support the adoption of molecular diagnostics as a frontline tool for managing BRD and contribute to improved disease surveillance, targeted interventions, and enhanced animal health outcomes in the cattle industry.

## Data Availability

The original contributions presented in the study are included in the article/Supplementary material, further inquiries can be directed to the corresponding author/s.

## Footnotes

^1^ https://blast.ncbi.nlm.nih.gov/Blast.cgi
