# Supplementary material for: Field-validated multiplex RT-qPCR for simultaneous detection of bovine respiratory syncytial virus and bovine parainfluenza virus-3 in bovine respiratory samples
Source: Front Vet Sci. 2025 Aug 29;12:1645647. doi: 10.3389/fvets.2025.1645647 (PMC12426054; doi:10.3389/fvets.2025.1645647)
Supplement: Supplementary file 1 [file Data_Sheet_1.pdf]

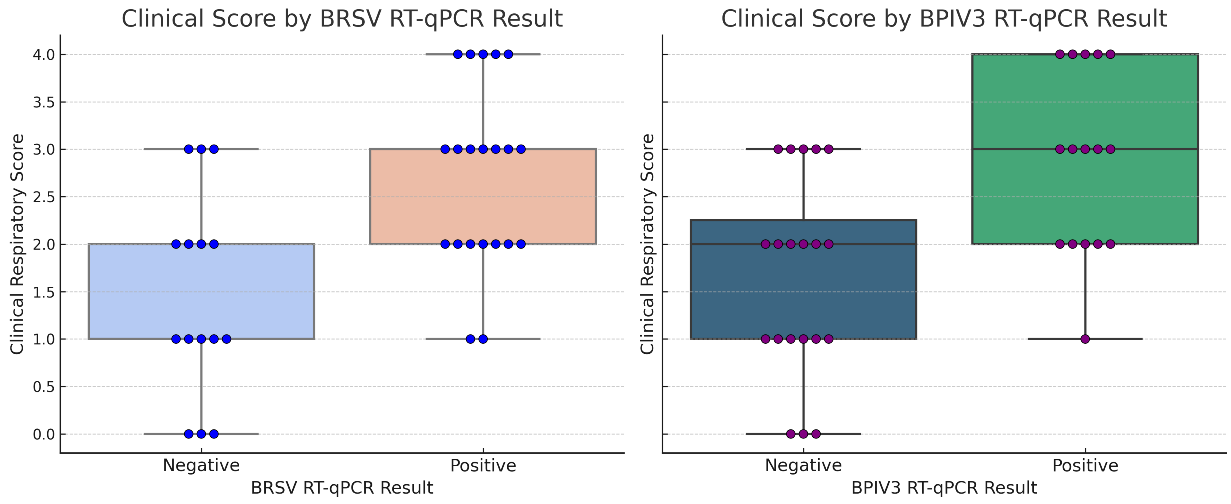


## Supplementary Figure 1. Clinical Respiratory Scores by BRSV and BPIV3 RT-qPCR Results

Box-and-swarm plots showing the distribution of clinical respiratory scores in animals tested for BRSV (left) and BPIV3 (right) by RT-qPCR. Scores range from 0 (no signs) to 4 (severe disease), shown in 0.5 score increments. Higher clinical scores were more frequently observed in RT-qPCR-positive animals. Swarm dots represent individual samples.

**Supplementary Table 1. GenBank Accessions Used for Primer and Probe Design**

| Virus | Accession Number | Gene Region | Source | Date Accessed |
|---|---|---|---|---|
| BRSV | AF295544.1 | N gene | USA/2000 | 2023-04-15 |
| BRSV | OP137030.1 | N gene | China/2022 | 2023-04-15 |
| BRSV | KY753470.1 | N gene | Italy/2017 | 2023-04-15 |
| BRSV | OM965703.1 | N gene | Japan/2022 | 2023-04-15 |
| BRSV | AF054667.1 | N gene | Denmark/1998 | 2023-04-15 |
| | | | | |
| BPIV3 | NC_002161.1 | NP gene | USA/2000 | 2023-05-11 |
| BPIV3 | Y00114.1 | NP gene | Japan/1987 | 2023-05-11 |
| BPIV3 | MH552577.1 | NP gene | China/2018 | 2023-05-11 |
| BPIV3 | KU255486.1 | NP gene | USA/2015 | 2023-05-11 |
| BPIV3 | OQ349373.1 | NP gene | Sweden/2023 | 2023-05-11 |

**Supplementary Table 2. Detection of BRSV and BPIV3 in Diluted Pooled Samples**

| Virus | Sample ID | Dilution Level | Genome Copies per Reaction | Ct Value | Detection |
|---|---|---|---|---|---|
| BRSV | BRSV-Pos1* | Neat | 100000.00 | 17.20 | Yes |
| BRSV | BRSV-Pos1 | 1:10 | 10000.00 | 21.50 | Yes |
| BRSV | BRSV-Pos1 | 1:100 | 1000.00 | 27.40 | Yes |
| BRSV | BRSV-Pos1 | 1:1000 | 10.00 | 33.90 | Yes |
| BPIV3 | BPIV3-Pos1[@] | Neat | 100000.00 | 18.10 | Yes |
| BPIV3 | BPIV3-Pos1 | 1:10 | 10000.00 | 22.30 | Yes |
| BPIV3 | BPIV3-Pos1 | 1:100 | 1000.00 | 28.20 | Yes |
| BPIV3 | BPIV3-Pos1 | 1:1000 | 10.00 | 34.70 | Yes |

*BRSV-Pos1 contained positive sample# 8233 pooled into negative sample# 2884, 3100, and 4180

[@]BPIV3-Pos1 contained positive sample# 3472 pooled into negative sample# 2884, 3100, and 4180.

**Supplementary Table 3. Standard Curve Ct Values for BRSV and BPIV3**

| Virus | Genome Copies (Log10) | Ct Replicate 1 | Ct Replicate 2 | Ct Replicate 3 |
|---|---|---|---|---|
| BRSV | 7 | 14.30 | 14.50 | 14.70 |
| BRSV | 6 | 17.10 | 17.20 | 17.30 |
| BRSV | 5 | 20.00 | 20.10 | 20.20 |
| BRSV | 4 | 23.40 | 23.50 | 23.60 |
| BRSV | 3 | 26.80 | 26.70 | 26.90 |
| BRSV | 2 | 30.20 | 30.30 | 30.10 |
| BRSV | 1 | 33.70 | 33.80 | 33.60 |
| | | | | |
| BPIV3 | 7 | 17.20 | 17.30 | 17.40 |
| BPIV3 | 6 | 20.00 | 20.10 | 20.20 |
| BPIV3 | 5 | 24.30 | 24.40 | 24.50 |
| BPIV3 | 4 | 28.70 | 28.80 | 28.60 |
| BPIV3 | 3 | 32.50 | 32.60 | 32.40 |
| BPIV3 | 2 | 36.70 | 36.80 | 36.60 |
| BPIV3 | 1 | 40.00 | 40.10 | 39.90 |

**Supplementary Table 4. Individual Ct Values from Standard Curve and Reproducibility Experiments**

| Virus | Dilution (Genome Copies) | Replicate 1 | Replicate 2 | Replicate 3 | Mean Ct | SD |
|---|---|---|---|---|---|---|
| BRSV | 1E+07 | 14.30 | 14.50 | 14.70 | 14.50 | 0.20 |
| BRSV | 1E+06 | 17.10 | 17.20 | 17.30 | 17.20 | 0.10 |
| BRSV | 1E+05 | 20.00 | 20.10 | 20.20 | 20.10 | 0.10 |
| BRSV | 1E+04 | 23.40 | 23.50 | 23.60 | 23.50 | 0.10 |
| BRSV | 1E+03 | 26.80 | 26.70 | 26.90 | 26.80 | 0.10 |
| BRSV | 1E+02 | 30.20 | 30.30 | 30.10 | 30.20 | 0.10 |
| BRSV | 1E+01 | 33.70 | 33.80 | 33.60 | 33.70 | 0.10 |
| BPIV3 | 1E+07 | 17.20 | 17.30 | 17.40 | 17.30 | 0.10 |
| BPIV3 | 1E+06 | 20.00 | 20.10 | 20.20 | 20.10 | 0.10 |
| BPIV3 | 1E+05 | 24.30 | 24.40 | 24.50 | 24.40 | 0.10 |
| BPIV3 | 1E+04 | 28.70 | 28.80 | 28.60 | 28.70 | 0.10 |
| BPIV3 | 1E+03 | 32.50 | 32.60 | 32.40 | 32.50 | 0.10 |
| BPIV3 | 1E+02 | 36.70 | 36.80 | 36.60 | 36.70 | 0.10 |
| BPIV3 | 1E+01 | 40.00 | 40.10 | 39.90 | 40.00 | 0.10 |
